# Long-term outcome in pediatric surgical bypass grafting after traumatic injury and tumor resection: retrospective cohort analysis

**DOI:** 10.1038/s41598-021-94971-2

**Published:** 2021-08-11

**Authors:** Stephanie Kampf, Madeleine Willegger, Christopher Dawoud, Gerhard Fülöp, Philipp Lirk, Andrea Willfort-Ehringer, Christoph Neumayer, Bernd Gollackner

**Affiliations:** 1 Division of Vascular Surgery, Department of Surgery, Medical University Vienna, Vienna, Austria; 2 Department of Orthopedics and Trauma-Surgery, Medical University of Vienna, Vienna, Austria; 3 Austrian Public Health Institute, Gesundheit Österreich GmbH, Vienna, Austria; 4 Department of Anesthesiology, Perioperative and Pain Medicine, Brigham and Women’s Hospital, Harvard Medical School, Boston, MA USA; 5 Division of Angiology, Department of Internal Medicine, Medical University of Vienna, Vienna, Austria

**Keywords:** Outcomes research, Paediatric research

## Abstract

Vascular bypass surgery in children differs significantly from adults. It is a rarely performed procedure in the setting of trauma and tumor surgery. Besides technical challenges to reconstruct the small and spastic vessels, another concern in bypass grafting is the adequate limb length growth over time. The primary aim of this study was to assess long-term outcome after pediatric bypass grafting, in a single academic center, focusing on potential effects on limb development. In this retrospective cohort analyses we included all pediatric patients undergoing vascular bypass grafting at our department between 2002 and 2017. All patients ≤ 18 years suffered a traumatic injury or underwent a tumor resection of the lower or upper limb. The youngest female patient was 0.4 years, the youngest male patient was 3.5 years. During the observation period, 33 pediatric patients underwent vascular repair, whereby 15 patients underwent bypass grafting. Median overall follow-up was 4.7 years (IQR ± 9). 8 patients (53%) had a traumatic injury (traumatic surgery group) and 7 patients had a planned orthopedic tumor resection (orthopedic surgery group). In 13/15 (87%) a great saphenous vein (GSV) graft and in 2/15 (13%) a Gore-Tex graft was used for bypassing. Both Gore-Tex grafts showed complete occlusion 12 and 16 years after implantation. No patient died in the early postoperative phase (< 30 days), however 3/7 (43%) in the orthopedic group died during follow-up. Revision surgery had to be performed in 1/15 (7%) patients. A functional use of the extremity was reported in all patients. Normal limb length growth according to the contralateral site, and therefore bypass growth, could be documented in 14/15 patients. Children are surgically challenging. In our study, surgery by a specialized vascular surgery team using GSV grafts led to adequate limb length and bypass growth, and we observed no functional restrictions.

## Introduction

Vascular injury due to trauma or oncologic resection requiring bypass grafting is a rare condition in the pediatric population. Only about 0.6 to 1.4% of all pediatric injuries are known to affect the vascular system^1,2^. Notably, pediatric patients with traumatic vascular injury have an improved adjusted mortality rate compared to adults^3^.

In addition, differences in outcome of the pediatric patients is attributed to several reasons, most prominently technically challenging revascularizations because of a small diameter of the vessel and extensive vasospasm rate, a lower long-term patency rate and possible long-term effects on normal limb growth (limb length discrepancy, bone age restriction, ischemic contractures). Limited long-term data are available on developmental impairment and limb growth after pediatric revascularization^4–7^.

Surgical repair, which is in most cases manageable by open surgical repair, includes primary repair, venous bypass grafting with great saphenous vein (GSV), or synthetic bypass grafting^8–10^. Endovascular management in pediatric trauma is much rarer^11^.

Management of pediatric vascular trauma is characterized by a low incidence and therefore limited clinical experience. Similarly, while there is a significant amount of data describing the management of vascular injury in both adult civilian and military patients, pediatric vascular trauma is mostly described after endovascular interventions^8,9^. Vascular reconstruction in pediatric patients after tumor resection accounts for only 0–23%^10–13^. Only few case series considering long-term outcome in children undergoing pediatric bypass grafting in the developed countries exist^14–16^.

Therefore, surgical management of these pathologies is based on extrapolation of data retrieved from adult vascular injuries^17^, and no explicit consensus guidelines on reconstruction in pediatric vascular trauma patients have yet been defined.

The aim of this study was to analyze long-term outcome of pediatric bypass grafting in a single academic center, focusing both on graft physiology, and potential developmental impact.

## Methods

### Patient characteristics

This study represents a retrospective cohort analysis of pediatric patients who underwent vascular bypass grafting by an experienced team of vascular surgeons at our department between 2002 and 2017.

All data regarding demographics, operative techniques and outcome were collected from the in-hospital database registry in collaboration with the Department of Orthopedics and Trauma-Surgery. All patients ≤ 18 years who suffered a traumatic injury or vascular surgery due to a planned orthopedic extremity tumor resection were included in this study. 33 patients underwent vascular repair, a total of 15 patients fulfilling the criteria could be identified.

This study was approved by the local institutional review board (ethics committee Medical University of Vienna, EK Nr: 1025/2018) and was performed in accordance with the principles of the Declaration of Helsinki. Informed consent was waived by ethics committee of the Medical University of Vienna.

### Vascular surgery

In all patients, an open surgical procedure was performed using either well-spatulated continuous or multiple single suture technique, depending on the diameter of the vessel and age of the patient.

For bypass grafting, autogenous (13/15) or artificial (2/15) material was used to perform the surgery in the upper and lower limb. Time to revascularization was determined measuring the time from first contact in the emergency department, to end of intraoperative suturing in the traumatic surgery group (ischemic time) or direct intraoperative clamping to revascularization in the oncologic surgery group.

Furthermore, technical success and bypass patency rate without stenosis (> 30%) was assessed.

### Perioperative results and outcome

Antiplatelet therapy and anticoagulation was assessed from medical reports during hospitalization, as well as follow up examinations.

Furthermore, mortality rate, infection rate, rate of revision surgery (< 30 days), amputation-free survival and limb-length disparities, bypass growth, and limb growth restriction during follow-up period were recorded.

Outcome analysis of the bypass included either duplex ultrasonography (DUS) or rarely computer tomographic angiography (CTA) or Magnet resonance angiography (MRA) due to oncologic investigations. Clinical examinations were carried out 1 month, 6 months, 1 year and annually after surgery. For clinical follow-up, an intact distal pulse, capillary refill, color, warmth, functionality, longitudinal growth (height) and length of the extremities were determined. Limb length disparities were determined by comparing length of both sides and gait pattern.

### Statistics

Demographic data for patient characteristics were evaluated via contingency tables. Metric data were expressed as median with IQR and were analyzed by using Mann Whitney U test.

A p-value < 0.05 was considered significant. For statistical analyses SPSS^®^ version 25.0 software (SPSS Inc., Chicago, IL, USA) was used.

## Results

At our department, 33 pediatric patients underwent vascular repair between 2002 and 2017.

Six patients received a primary vascular repair with a patch plasty due to complications after extracorporeal membrane oxygenation (ECMO) or angiography; 12 patients received a flap surgery after tumor resection with primary vascular reconstruction; and 15 patients received a bypass graft surgery.

### Patient characteristics

Patient characteristics are shown in Table 1. In brief, 15 pediatric patients undergoing oncologic resection or vascular trauma were identified at our department.

**Table 1:** Trauma vs. oncologic surgery group.

	Trauma	Oncology
	Median (± IQR)	Median (± IQR)
Patient number	8	7
Age (years)	11.6 (± 12)	11.8 (± 5.2)
Gender (female:male)	1:1.7	1:0.8
BMI	18.6 (± 3.18)	17.3 (± 4.7)
Follow-up	8.9 (± 14.5)	4.7 (± 1.7)
**Extremity**		
Upper	3	0
Lower	5	7
Time (hours): contact—revascularization	5 (± 5)	0.5 (± 0.2)

Within the study cohort, 8 patients (53%) underwent vascular bypass graft surgery due to a traumatic injury to the lower or upper limb and 7 patients (47%) due to a planned orthopedic tumor resection of the lower or upper limb.

Overall median age was 11.8 years (IQR: ± 6) at time of surgery, with no statistically significant differences between groups (p = 0.817). The youngest female patient was 0.4 years, the youngest male patient was 3.5 years.

Follow up was shorter in the orthopedic surgery group (trauma surgery group: 8.9 years [± 14.5], orthopedic surgery group: 4.7 years [± 1.7]; p = 0.379). Two patients were excluded from follow up analysis for early death during follow-up period due to tumor progression or infection.

### Vascular surgery (Table 2)

**Table 2:** Patient characteristics–vascular surgery.

Patient number	Indication	Gender	Age at surgery (years)	Graft	Graft type	Suture technique	Operation type	Revision surgery (< 30 days)	LOS (days)
1	Trauma	female	0.4	Artifical	PTFE	Interrupted	Femoro-popliteal	No	n.a.
2	Trauma	Male	3.5	Autograft	GSV	Interrupted	Illio-femoral	No	6
3	Trauma	Female	7.6	Autograft	GSV	Interrupted	Brachial-brachial	No	6
4	Trauma	Male	11.6	Artifical	PTFE	Running	Subclavian-brachial	No	9
5	Trauma	Male	15.0	Autograft	GSV	Running	Ilio-femoral	No	8
6	Trauma	Male	15.5	Autograft	GSV	Running	Brachial-brachial	No	24
7	Trauma	Female	17.7	Autograft	GSV	Running	Femoro-popliteal	No	12
8	Trauma	Male	18.0	Autograft	GSV	Running	Femoro-distal	Yes^a^	7
9	Osteosarcoma	Male	9.3	Autograft	GSV	Interrupted	Illio-femoral	No	14
10	Neuroblastoma	Male	9.5	Autograft	GSV	Interrupted	Femoro-femoral	No	43
11	Osteosarcoma	Male	10.8	Autograft	GSV	Running	Femoro-distal	No	9
12	Osteosarcoma	Female	11.8	Autograft	GSV	Interrupted	Femoro-popliteal	No^b^	63
13	Osteosarcoma	Female	12.1	Autograft	GSV	Interrupted	Femoro-popliteal	No	31
14	Ewing's sarcoma	Female	14.7	Autograft	GSV	Interrupted	Illio-femoral	No	9
15	Osteosarcoma	Female	15.3	Autograft	GSV	Interrupted	Illio-femoral	No	13

*gsv* great saphenous vein.

^a^Postoperative bleeding treated by interrupted suturing.

^b^Postoperative ischemia treated by fasciotomy.

All patients underwent surgical bypass grafting. Median time from first contact to revascularization was 5 h (± 5) in the traumatic surgery group and 0.5 h (± 0.2, p = 0.002) in the planned orthopedic surgery group. Median length of stay (LoS) was 8 days^6–12^ for the traumatic surgery group and 14 days (9–43) for the orthopedic surgery group. Although the length of stay was depending on the extent of additional trauma and further treatment (e.g.: chemotherapy, radiation therapy) (Table 2).

A surgical bypass grafting to the upper limb was performed 3/8 (38%) times in the traumatic surgery group and 0/7 times in the orthopedic surgery group. In these patients a subclavian-brachial bypass was performed once and a brachial-brachial bypass twice. 5/8 patients in the traumatic surgery group suffered a trauma to the lower limb, an illio-femoral bypass was performed twice, a femoro-popliteal bypass twice and a femoro-distal bypass once.

In the oncologic surgery group, bypass grafting was only performed to the lower limb: 1 femoro-femoral bypass, 3 illio-femoral bypasses, 2 femoro-popliteal bypasses and 1 femoro-distal bypass.

**Figure 1:**
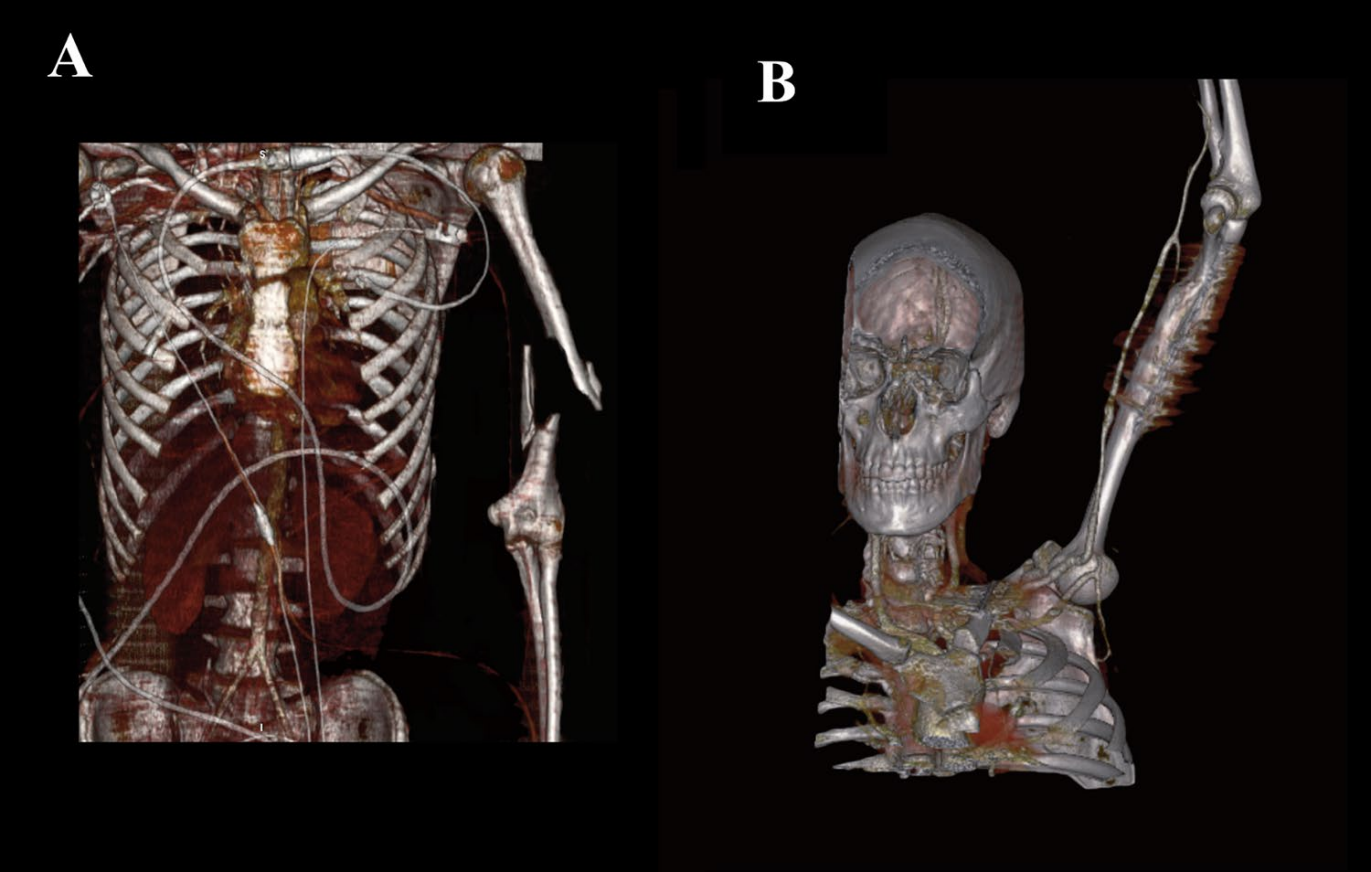
Pre-, and postoperative 3D reconstruction of a patient with a dissection of the left axillary and brachial artery. (**A**) Preoperative imaging of the patient. (**B**) Postoperative imaging 11 years after a GSV brachial-brachial bypass.

**Figure 2:**
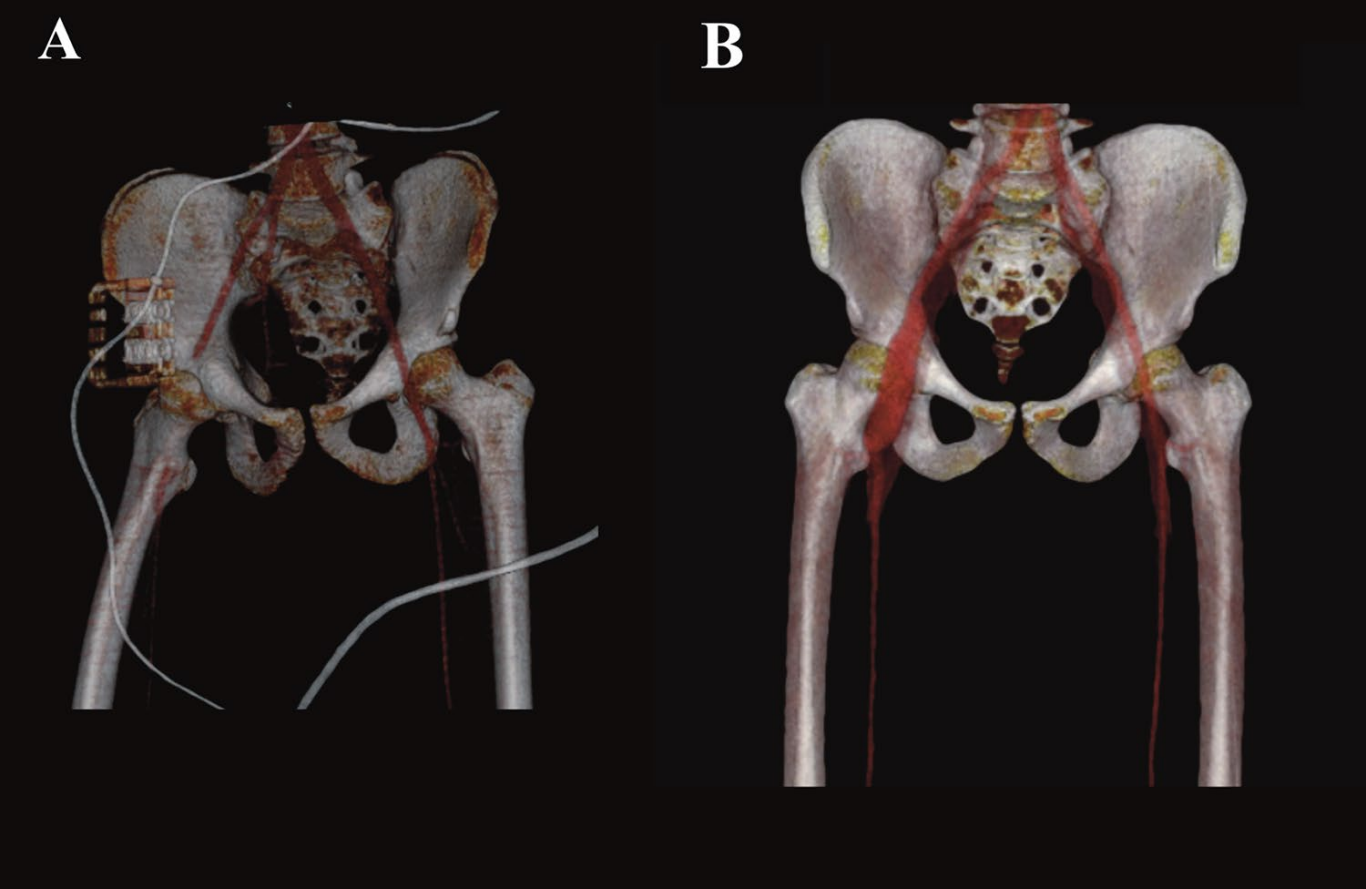
Pre- and postoperative 3D reconstruction of a patient after a bike accident and dissection of the right femoral artery. (**A**) Preoperative imaging of the patient. (**B**) Postoperative imaging 3 years after a GSV ilio-femoral bypass (*graft dilation up to 22 mm).

For an autologous bypass, the great saphenous vein (GSV) was used in all patients, 13/15 (87%). The youngest patient with a GSV graft was 3.5 years at time of surgery (selected samples in Figs. 1, 2).

In two patients within the traumatic surgery group (25%), GSV could not be used and a PTFE graft was used: once as subclavian-brachial bypass (Goretex^®^ Vascular Graft, ring-reinforced 6 mm, Gore & Associates Inc., Newark, DE, USA) and once as femoro-popliteal bypass (IMPRA Carboflo™ Vascular Graft, 6 mm, IMPRA Inc., Tempe, AZ). PTFE grafts were only used if no convenient vein was available or present vein diameter was insufficient.

In both patients no infection occurred postoperatively. An occlusion of the subclavian-brachial bypass was detected 16 years after Gore-Tex implantation, but due to good collateralization and no motoric or sensory deficits, no surgical intervention was required. The youngest patient (0.4 years) with an artificial graft (IMPRA Carboflo™, 6 mm) showed stenosis, caused by thrombotic formations, which preserved flow in DUS 3 years after surgery. An occlusion was detected by CTA 12 years after implantation, but no revision surgery was required, again due to good collateralization and no deficiencies.

### Perioperative results and outcome (Table 3)

**Table 3:** Patient characteristics—outcome.

Patient number	Infection	ICU	Antiplatelet therapy/anticoagulation	1st control (1 month)			2nd control (6 month)			3rd control (1 year)			Last control			Follow up (years)	Death
				Clinical	Limb length disparty	DUS/CTA/MRA	Clinical	Limb length disparty	DUS/CTA/MRA	Clinical	Limb length disparty	DUS/CTA/MRA	Clinical	Limb length disparity	DUS/CTA/MRA		
1	No	No	None	Pulse	None	DUS	Pulse	None	DUS	Pulse	None	DUS	No pulse^a^	None	CTA—complete occlusion	23.2	No
2	No	Yes	6 month: aspirin 50 mg, enoxaparin 20 mg 1-0-0	Pulse	None	DUS	Pulse	None	DUS	Pulse	n.a.	n.a.	Pulse	None	DUS	0.7	No
3	No	No	6 month: T-ASS 50 mg	Pulse	None	DUS	Pulse	None	DUS	Pulse	n.a.	n.a.	Pulse	None	DUS	0.5	No
4	No	No	Long-term: Aspirin 50 mg	Pulse	None	DUS	Pulse	None	DUS	Pulse	None	CTA	No pulse^b^	None	CTA—complete occlusion	16.7	No
5	No	No	6 month: aspirin 100 mg, enoxaparin 40 mg 1-0-1	Pulse	None	CTA	Pulse	Yes (1 cm)	DUS	Pulse	Yes (1 cm)	DUS	Pulse	None Distraction osteogenesis^c^	CTA—aneurysmatic dilatation (22 mm)	2.6	no
6	No	Yes	6 month: aspirin 100 mg, enoxaparin 40 mg 1-0-1	Pulse	None	CTA	Pulse	None	DUS	Pulse	None	n.a.	Pulse	None	CTA	10	No
7	No	No	Long-term: aspirin 50 mg	Pulse	None	DUS	Pulse	None	DUS	Pulse	None	DUS	Pulse	None	DUS	7.9	No
8	No	No	Long-term: aspirin 100 mg, enoxaparin 80 mg 1-0-0	Pulse	None	MRA	Pulse	None	CTA—dissection of ATP (PTA + stent)	Pulse	None	DUS	Pulse	None	CTA—aneurysmatic dilatation (18 mm)	12.7	No
9	No	No	n.a.	Pulse	None	MRA	Pulse	None	n.a.	Pulse	None	DUS	Pulse	None	n.a.	4.7	Yes
10	Yes^d^	Yes^d^	n.a.	Pulse	n.a.	n.a.	n.a.	n.a.	n.a.	n.a.	n.a.	n.a.	n.a.	n.a.	n.a.	0.1	Yes^d^
11	No	No	Aspirin 50 mg	Pulse	Yes (1 cm)	MRA	Pulse	Yes (1 cm)	DUS	Pulse	Yes (2 cm)	DUS	Pulse	Yes (2 cm)	n.a.	6.0	No
12	No	Yes	Long-term: aspirin 100 mg 3 month: enoxaparin 60 mg 1-0-0	Pulse	Yes (1 cm)	MRA	Pulse	Yes (15 cm rotation plasty)	DUS	Pulse	Yes (15 cm rotation plasty)	MRA	Pulse	Yes	n.a.	3.7	No
13	Yes^c^	No	3 month: enoxaparin 20 mg 1-0-1	Pulse	Yes (amputation)	CTA	n.a.^c^	n.a.^c^	n.a.^c^	n.a.^c^	n.a.^c^	n.a.^c^	n.a.^c^	n.a.^c^	n.a.^c^	4.7	No
14	No	Yes	3 month: enoxaparin 40 mg 1-0-0	Pulse	Yes (inner hemipelvectomy 15 cm)	CTA	Pulse	n.a.	CTA	Pulse	n.a.	n.a.	Pulse	n.a.	DUS	5.8	Yes
15	No	Yes	3 month: aspirin 50 mg, enoxaparin 20 mg 1-0-0	Pulse	No	CTA	Pulse	No	DUS	Pulse	No	n.a.	Pulse	No	CTA	4.5	No

Graft is patent by the imaging modality stated in the chart, unless otherwise noted.

^a^Occlusion of the artificial bypass graft 12 years after surgery, with good collateralization.

^b^Occlusion of the artificial bypass graft 16 years after surgery, with good collateralization.

^c^Amputation due to orthopedic implant infection, 2 months after surgery.

^d^Death due to septic event.

All patients received anticoagulation with low molecular weight heparin during the hospital stay at therapeutic dosage. Antiplatelet pharmacotherapy with acetylsalicylic acid (50 mg) was initiated in 7/8 patients from the traumatic surgery group and was at least continued for 6 months. However, due to necessity of adjuvant chemotherapy only 3/7 (43%) patients received postoperative antiplatelet therapy in the orthopedic surgery group. Antiplatelet therapy in this group was continued for less than 3 months. One patient suffered from coagulation disorder and had to continue intake infinitely.

Except for one polytrauma patient, all patients in the traumatic group were treated at an intermediate care unit, whereas 4/7 (57%) patients in the orthopedic surgery group had to be treated at an intensive care unit due to an extensive orthopedic tumor resection.

One patient (1/15, 7%) in the traumatic cohort had to undergo revision surgery of the graft due to a postoperative bleeding. In another patient of the orthopedic cohort, fasciotomy due to reperfusion injury was indicated. On postoperative day one, the patient presented with swelling of the lower leg due to a prolonged operating time based on the size of the mass (> 8 h).

Surgical site infection occurred in 2/7 patients in the orthopedic surgery group. One patient suffered an infection of Enterobacter cloacae and the other patient suffered an infection of Saccharomyces cerevisiae during chemotherapy, resulting in systemic infection and death of the patients. In the traumatic surgery group, no patient had any surgical site infection.

No patient died in the traumatic surgery group, however 3/7 (43%) patients died in the orthopedic surgery group. Two cases were associated with septic events associated with immunosuppression during chemotherapy, and one patient died due to tumor progression.

The median follow-up in traumatic surgery group was 8.9 years (± 14.5), overall follow-up was 4.7 years (± 9). No patient had to undergo amputation due to an ischemic event.

2/15 patients showed aneurysmatic dilation during follow-up CTA, with no intervention (Table 3).

**Figure 3:**
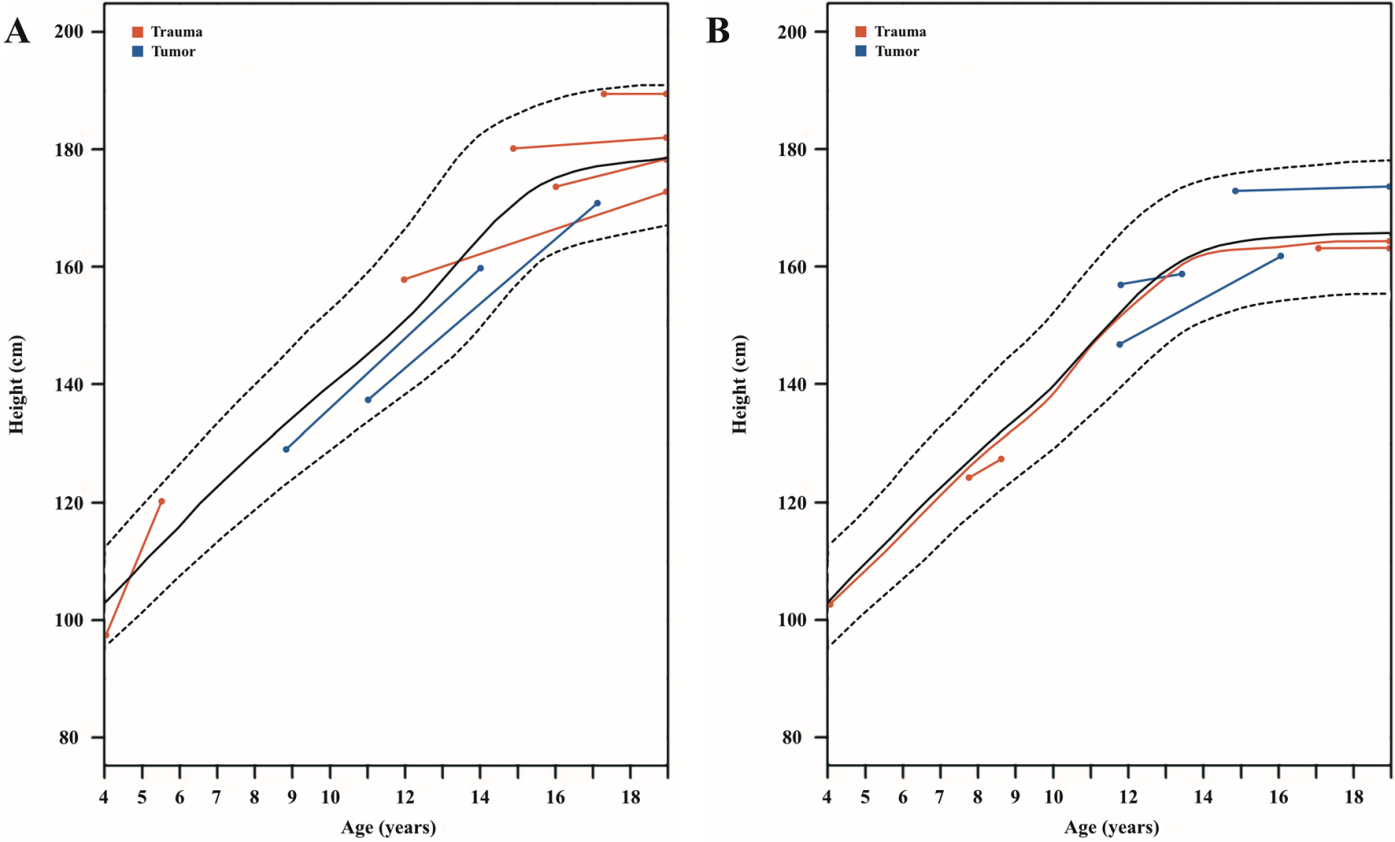
Patients’ height progress during follow-up, according to the new austrian height and proportion percentile curve^34^. (**A**) Percentile progress of male patients’ height (red: trauma patients, blue: tumor patients). (**B**) Percentile progress of female patients’ height (red: trauma patients, blue: tumor patients). *Patient number 1 is included from 4 to 19 years.

One patient of the traumatic surgery group (age 15.0 years) had to undergo a distraction osteogenesis due to limb length discrepancy of 5 cm 2 year after bypass grafting. However, this length discrepancy was suspected to be associated with the polytraumatic event and an early bone growth stop and not due to an insufficient supply of the vascular bypass. In all other patients, a functional use of the extremity involved was reported. A normal limb length growth according to the opposite side, and bypass growth in DUS, could be documented. In the youngest male patient with an GSV graft, a normal limb length, without side discrepancy was seen 2 years after surgery (3.5 years height: 94 cm vs. 5 years height: 120 cm) (Fig. 3).

In one patient of the orthopedic surgery group, an amputation surgery due to orthopedic implant infection 2 months after surgery had to be performed.

## Discussion

Our single center cohort series describes 15 patients requiring revascularization after oncologic resection or trauma to the lower or upper limbs with a follow up period of up to 14 years (median: 4.7 ± 9 years). The results of follow-up controls revealed new aspects for this rarely surveyed group, as all included patients underwent an immediate open surgical approach, without endovascular stenting or bridging. We could demonstrate for the first time, that even small children show adequate bypass and limb length growth with no functional restrictions.

Considering amputation free survival and functional status after revascularization, we were able to determine venous interposition grafts to show continuously good long-term results.

Only few cohort studies of pediatric bypass grafting with a low number of patients (max. 23 patients) have been published up to now^17–22^. In trauma patients, vascular injuries in the pediatric field represent a particular challenge, due to a low incidence and no explicitly declared treatment strategies to date^14,15^. Penetrating mechanisms of injury are more common than blunt injuries^2^. A small caliber of the vessels and the need for further growth has to be considered before surgical intervention. However, data on long-term outcome as functional limb status and patency rate is limited^18,19^.

In our case series, synthetic conduits PTFE (Polytetrafluoroethylene) showed a reduced long-term patency and were only used if the vein was not suitable for bypassing. Only two patients of our cohort had a synthetic graft repair and both patients showed complete occlusion 12 and 16 years after surgery. To the best of our knowledge no series of synthetic bypass grafting in children has been published. Reviewing the literature considering adult synthetic bypass grafting, a higher rate of infection and a reduced long-term patency could be demonstrated^20^. Although, due to the very small number of patients with artificial bypass grafts in our study, we cannot conclude, that synthetic bypass grafting is inferior to venous bypass grafting.

In our series, none of the two patients with synthetic grafts had a postoperative wound infection. Two patients with an autologous graft had a serious infection, which could be traced back to a reduced immunologic response after chemotherapy, leading to death 1 month and 6 months after surgical revascularization^21,23^. However, in combined series of adult and pediatric patients with orthopedic tumors, infection was one of the main reasons for failure of vascular reconstruction^10^.

A major complication in pediatric vascular surgery is extremity growth arrest after bypass grafting when there is no bypass growth, which might require revision surgery or therapeutic vein graft dilatation^24,25^. However, in our series no secondary therapeutic graft dilatation during long-term follow-up had to performed. All patients, except one showed adequate limb length growth and therefore bypass growth. Interrupted suture technique is recommended by Meagher et al.^26^ and has shown adequate short-term results. In contrast Whitehouse et al.^27^ recommended only generous spatulation independent of continuity or interruption of suture.

In recent literature, the patency rate of grafts sutured in continuous technique is described as equivalent to interrupted suture technique. It is a reliable technique requiring shorter total operative time, if performed by an experienced surgeon. Furthermore, this technique can be used for vessels with diameters larger than 1 mm^28^. In our patients either well spatulated continuous or multiple single suture technique was used depending on the diameter of the vessel and intraoperative decision of the surgeon, leading to comparable results. No patient showed a functional disability or chronic wounds during follow up. No patient required amputation or revision surgery due to occlusion of the bypass graft.

To the best of our knowledge there are only two more series describing explicit long-term pediatric bypass results. The University of Michigan series, including 14 pediatric patients with bypasses, with a mean follow up of 8 years and an excellent long-term patency rate of autogenous grafts. Though, the Michigan series features mostly delayed revascularization with an average time of 5.7 years after injury and is therefore not fully comparable to our retrospective analysis. Secondly the Indiana University School of Medicine series describing 23 cases of immediate pediatric peripheral revascularization in a long-term follow up period of 3.5 years^14,15^. At our center, the median overall follow-up period was 4.7 years (± 9), respectively 8.9 years (± 14.5) for the traumatic surgery group. Vascular surgeons and orthopedic-trauma colleagues are available 24/7. For traumatic incidents, our orthopedic-trauma colleagues and anesthesiologists are providing initial examination, medical treatment and diagnostics (CTA). Definite treatment strategies are discussed in an interdisciplinary approach. The majority of trauma patients had associated bone fractures requiring immediate revascularization. The median time from initial injury pattern to completion of revascularization was 5 h. For the oncologic surgery group, there was an obviously faster time of revascularization. This short time in both groups may be favorable for good long-term results and also shows the importance of an interdisciplinary collaboration.

Reviewing the literature, historically, major vascular trauma in pediatric patients up to 6 years were suggested to preferably receive conservative non-invasive treatment due to poor reported outcome^29,30^. Conversely, recent publications underline the possibility of a successful revascularization and therefore show equal findings to our case series^14,15^.

In our patient cohort, all patients were in need of acute revascularization for limb preservation. All patients, except two, were older than 4 years of age. Follow up of our youngest male patient showed excellent clinical and duplex-sonographic results 2.5 years after reconstruction. The patient showed no signs of growth restriction (height at surgery: 97 cm. vs. height 2.5y follow-up: 120 cm) or limb length discrepancy.

In contrast to vascular injuries with long-term follow up^14,15^, little is known about the limb salvage rate in pediatric patients undergoing a revascularization after an orthopedic tumor resection. Although only extrapolation of data from adult and pediatric small case series is available, revascularization has been described as an effective method to preserve the limb^10–13^. This appears, to be in accordance with our data, as only in one case of orthopedic implant infection, an amputation had to be performed. However, especially in pediatric cases with malignancies in need of postoperative chemotherapy, synthetic grafts should be avoided due to a possible bacterial colonization based on an induced immunodeficiency.

In our opinion, successful bypass grafting in pediatric patients can be performed by any experienced surgeon if accurate suture techniques are applied and GSV grafts are used preferably. Pediatric vessels show an extensive rate of perioperative and postoperative vasospasm due to surgical handling; therefore, an adequate distal pulsation may not occur instantly after vascular clamp release. After surgical bypass grafting adult patients should maintain on antiplatelet therapy for at least 3 month^31^. There are no distinct guidelines concerning anticoagulation or antiplatelet therapy in the pediatric patients after bypass grafting. As in our series patients were kept on low antiplatelet therapy for 3 to 6 months, which may be one reason for the good patency rate in our cohort. Reye’s syndrome can result from children taking salicylates, but is known to be dose-dependent and normally occurs with high dosage of antiplatelet therapy (> 40 mg/kg). Our patients only received low-dose antiplatelet therapy (1–5 mg/kg) if indicated, therefore no patient was affected by Reye’s Syndrome. Though, the role of low-dose antiplatelet drugs in the management of pediatric bypass grafting should be addressed in further studies.

Vascular surgery is not a core competency in the Austrian orthopedic-trauma surgical or pediatric surgical fellowship^32^. In our cohort, a vascular surgery consultant was always part of the surgical team. Our high long-term patency rate of 93.3% is comparable to that of specialized level I pediatric trauma centers (87%)^33^. This underlines the necessity of an interdisciplinary collaboration.

### Limitations

The limitations of this study are inherent to the retrospective character and the limited number of cases in this study. However, the follow up of this small case series also brings up new aspects for this uncommon group of pediatric patients.

## Conclusion

Pediatric vascular bypass grafting requires a multidisciplinary approach. In our series all patients, except two with artificial grafts, demonstrated a satisfying functional and overall outcome, even in the youngest patients. Vascular surgery in pediatric patients is surgically challenging, but accurate suture techniques and GSV grafts lead to adequate bypass growth and limb length growth with no functional restrictions.
